# Mood profile in men and women of all ages is improved by leisure-time physical activity rather than work-related physical activity

**DOI:** 10.1186/s12889-024-17806-5

**Published:** 2024-02-21

**Authors:** Albertas Skurvydas, Natalja Istomina, Ruta Dadeliene, Daiva Majauskiene, Emilija Strazdaite, Ausra Lisinskiene, Dovile Valanciene, Aiste Barbora Uspuriene, Asta Sarkauskiene

**Affiliations:** 1 Department of Rehabilitation, Physical and Sports Medicine, Faculty of Medicine, Institute of Health Sciences, Vilnius University, 21/27 M.K. Ciurlionio St, 03101 Vilnius, Lithuania; 2 Institute of Health Sciences, Faculty of Medicine, Vilnius University, 21/27 M.K. Ciurlionio Street, 03101 Vilnius, Lithuania; 3 Education Academy, Vytautas Magnus University, K. Donelaicio Street 58, 44248 Kaunas, Lithuania; 4 Faculty of Law, Vilnius University, Sauletekio al. 9, 10221 Vilnius, Lithuania; 5 Department of Sports, Recreation and Tourism, Klaipeda University, Herkaus Manto Street 84, 92294 Klaipeda, Lithuania

**Keywords:** Leisure-time, Work-related, Occupational, Household, Physical activity, Mood, Vigour, Fatigue

## Abstract

**Background:**

The aim of our study was to determine how six mood indicators (vigour, fatigue, depression, anger, confusion, tension) depend on moderate to vigorous physical activity, walking time and sedentary time at work, after working hours and during leisure time, in men and women of different age groups.

**Methods:**

A total of 1,140 individuals aged 18 to 64 years participated in the study. The participants were enrolled in this cross-sectional survey using a snowball sampling method. An online questionnaire was shared through popular social networks and emails within the period October 2019 to June 2020. Mood responses were assessed using The Brunel Mood Scale-LTU. Physical activity was assessed using the long International Physical Activity Questionnaire. Descriptive analysis, a two-way analysis of variance, and linear regression analysis were used to interpret the data.

**Results:**

The survey results showed that vigour and fatigue correlated significantly only with leisure-time moderate to vigorous physical activity. The present results show a significant positive correlation between women and men moods and leisure-time moderate to vigorous physical activity, the length of time walking to work and back home, and negative correlation between moods and leisure-time sedentary behaviour. However, there was no significant correlation between moods and work-related moderate to vigorous physical activity and household moderate to vigorous physical activity, walking at work, and sitting duration at work.

**Conclusions:**

This study provides theoretical implications of the physical activity paradox, justifying the benefits of moderate to vigorous physical activity practiced in different circumstances. According to the regression analysis, exercising men in all age groups moved the most (had a higher moderate to vigorous physical activity level) during leisure time, the highest work-related moderate to vigorous physical activity was observed in men and women with lower education, and the highest household moderate to vigorous physical activity was observed in older age men and women living in rural areas. Clinicians and leaders at all levels of health care should consider the greater importance of leisure-time physical activity for mental health when choosing the most targeted physical activity recommendations for mood profile improvement in men and women of different age groups.

## Background

Mental health status is linked to both physical activity (PA) levels and to sedentary behaviour (SB) irrespective of PA. For example, Chekroud et al. collected data from more than 1.2 million adults in the United States and found that those who exercised had 43.2% fewer days of poor self-reported mental health than those who did not exercise [1]. A great number of epidemiological studies have also demonstrated that SB has a negative impact on physical and mental health, regardless of PA level [2]. The data of the observational study conducted by García-Soidán and co-authors showed that during the evaluated period of 20 years in the population of Spanish children, the increased use of electronic devices with screens changed childrens’ physical activity habits and encouraged a sedentary lifestyle, which is associated with certain emotional problems, such as anxiety, depression, and lower self-control [3]. Mood state is one of the indicators of psychological well-being and mental health [4], with 1 in 8 of the world’s population now living with a mental health disorder, most commonly depression and anxiety [5]. The state of mental health in Lithuania, with levels of subjective well-being lower than the European Union (EU) average [6], gives cause for concern. The Brunel Mood Scale (BRUMS-LTU), which was recently translated into Lithuanian, can be used as an indicator of mental health. Research has recently determined that mood depends on PA, sex, and age [7, 8]. The BRUMS has been often used in a mental health context to monitor well-being among cardiac rehabilitation patients and to evaluate population-level mental health in Brazil [9, 10]. The Brazilian researchers evaluated health and the quality of sleep but did not determine the level of PA. Both the Profile of Mood States (POMS) [11] and BRUMS have been used extensively in the domain of sport and exercise psychology to investigate the antecedents, correlations, and behavioural consequences of moods, in particular, the effects of moods on the performance and psychological well-being of athletes and exercisers [11, 12]. Moods have been shown to vary according to biological sex and gender identity, with men tending to report higher Vigour scores and lower Anger, Confusion, Depression, Fatigue, and Tension scores than women [7, 13]. Age has also been implicated in mood differences, with reported moods among adults tending to be more positive with increasing age [7].

Researchers have recently found a PA paradox, which revealed that leisure-time PA is more beneficial for health than work-related PA [14–21]. This PA paradox has been observed in regard to all-cause mortality [15, 16, 22], cardiovascular disease mortality [20], long-term sickness work absence [23], longevity [24], and systolic blood pressure [19]. Research has also determined that this paradox manifests differently in men and women [16, 24]. For example, a meta-analysis revealed an 18% higher risk of early all-cause mortality in men with high-level work-related PA than in men with low-level work-related PA [16]. However, such a correlation was not detected for women; in fact, an opposite trend was observed. Surveys of men and women in Norway showed that moderate to high work-related PA contributed to longevity in men; however, work-related PA did not increase longevity in women [24]. There is no agreement among researchers regarding the PA paradox [17, 18]. We therefore believe that the PA paradox has to be investigated depending on the type of work and on the intensity of work-related PA [21, 25].

The strength of our research in comparison with other similar studies lies in clarifying the PA paradox, taking into account the criteria not evaluated by other researchers in this context. Despite the studies cited above, there is still no clear consent on the PA paradox, because it depends not only on the structure of PA but also on the type of work, gender, age, the specificity of the recorded variables, and PA outside of working hours and during leisure time. We did not find any studies that investigated whether this paradox is reflected in mood indicators and whether it depends not only on moderate- and high-intensity PA but also on the duration of sedentary time and low-intensity PA, such as walking. On the basis of the PA paradox, we distinguished between healthy and unhealthy PA. Healthy PA is practised during leisure time, whereas unhealthy PA is occupational and includes physical work outside of working hours.

The aim of our study was to determine how six mood indicators (vigour, fatigue, depression, anger, confusion, tension) depend on moderate to vigorous PA (MVPA), walking time and sedentary time at work, after working hours and during leisure time, in men and women from different age groups. We also investigated the main factors that influence the choice of healthy and unhealthy PA.

## Methods

### Participants

A total of 1,140 individuals participated in the study. Three hundred nine (27.1%) identified as men (27.1%), and 831 (72.9%) identified as women (72.9%). Their ages ranged from 18 to 64 years. Sociodemographic data for the sample are provided in Table 1. Informed consent was obtained from all participants. All participants were informed about the goals of the study, the anonymity of their participation, and the option to cancel their participation at any time. Participants agreed to participate in the survey by filling in an online questionnaire. The survey was conducted in accordance with the Declaration of Helsinki, and the survey protocol was approved by Klaipėda University (Protocol No. STIMC-BTMEK-09).

### Survey design and procedure

The participants were enrolled in this cross-sectional survey using a snowball sampling method, a nonprobability sampling technique also called convenience sampling. We recruited our initial sample from available participants (personal and professional contacts: university students, colleagues, social media followers, and groups in social networks). These participants were asked to invite more participants and share the internet link for the survey with their friends and colleagues (i.e., potential participants aged 18 to 64 years). An online questionnaire was shared through popular social networks and emails within the period October 2019 to June 2020.

### Instruments

The Brunel Mood Scale-LTU (BRUMS-LTU), adapted from the Terry et al., was used [7]. Mood responses were assessed using the 24-item BRUMS-LTU. The scale has six subscales with four items each (i.e., Tension items: nervous, anxious, worried, panicky; Depression items: unhappy, miserable, depressed, downhearted; Anger items: bitter, angry, annoyed, energetic; Vigour items: energetic, active, lively, alert; Fatigue items: exhausted, tired, worn out, sleepy; and Confusion items: mixed up, muddled, uncertain, confused). The participants responded using a 5-point Likert scale (0 = not at all, 1 = a little, 2 = moderately, 3 = quite a bit, and 4 = extremely), with the total possible subscale scores ranging from 0 to 16. The time frame was ‘right now’ (e.g. ‘How do you feel right now?’). The 24 items condensed into six subscale scores were treated as scale variables. The BRUMS-LTU has demonstrated satisfactory internal consistency, with Cronbach’s α coefficients ranging from 0.74 to 0.90 for the six subscales.

PA was assessed by means of the long International Physical Activity Questionnaire (IPAQ) [26, 27]. This questionnaire covers four activity domains: work-related PA (paid employment as well as voluntary work), transportation PA, domestic PA, and recreational PA. The IPAQ items assess the frequency of PA (reported in number of days; “During the last 7 days, on how many days did you do...?’) and average duration of PA per day (reported in hours and minutes; ‘How much time did you usually spend on one of those days doing...? ’) in these specific PA domains. Total weekly PA was estimated by weighting the time spent on each intensity activity by its metabolic equivalent (MET) energy expenditure. The METs of vigorous, moderate-, and low-intensity activities were 8.0, 4.0, and 3.3, respectively.

Subjective health assessment was done by asking participants to answer the question ‘How would you evaluate your health condition in the past few months?’ The participants reported their health on a Likert-type scale (1 = poor, 2 = satisfactory, 3 = good, 4 = excellent). For further analysis, two categories were established: poor health and good health. The use of a self-report questionnaire is a reliable method that reflects the quality of women’s and men’s health.

### Data analysis

Descriptive statistics were used to present the data in a meaningful way and the normal distribution testing of continuous variables was done. Descriptive analysis, a two-way analysis of variance (ANOVA), and linear regression analysis were used to interpret the data. A univariate two-way ANOVA was performed to determine whether there was any correlation between the two independent variables and the dependent variable. If significant effects were found, Tukey’s post hoc adjustment was used for multiple comparisons within each repeated-measures ANOVA. The partial eta squared ($\eta_{\text{p}}^{2})$ value was estimated as a measure of effect size, and the β coefficient was estimated as the regression parameter. The reliability, or the internal consistency, of the questionnaires was measured by calculating the Cronbach’s α index. The statistical significance was defined as *p* < 0.05 for all tests. Statistical analyses were conducted using IBM SPSS Statistics software (version 22; IBM Corp., Armonk, NY, USA).

## Results

Information about the participants is presented in Table 1. There were more physically active men than women among the participants (*p* < 0.001). The average age of both groups was similar. Women had a lower body mass index (BMI) than men (*p* < 0.001). More women reported their health as excellent than men (*p* < 0.001).

**Table 1:** Sociodemographic information

Parameter	Gender		p-value
	Female *N* = 831 (72.9%)	Male *N* = 309 (27.1%)	
**Age, years**	41.9 (11.6)	40.1 (11.2)	0.12
**BMI, kg/m** ^**2**^	24.2 (4.5)	26.5 (4.9)	0.001
**Education**			
Not finished secondary education	0.7%	0.6%	> 0.05
Secondary education	7.8%	8.4%	
Vocational education and training	4.7%	4.0%	
Higher education (non-university)	7.9%	12.6%	
Higher education (university)	78.9%	74.4%	
**Sport**			
I don’t exercise	30.9%	17.4%	0.001
I’m in a professional sport	1.8%	5.8%	< 0.05
I exercise on my own	51.7%	61.8%	
I exercise in a gym/health centre	15.5%	15%	> 0.05
**Health**			
Excellent	15.5%	25%	0.001
Good	57.3%	54%	> 0.05
Satisfactory	24.3%	19%	
Poor	2.9%	2%	
**Type of job/vocation**			
Sedentary work	51.0%	43.4%	> 0.05
Working while seated/standing/walking at low intensity	28.2%	27.7%	
Heavy lifting, walking, intense work	19.7%	25%	
Heavy physical work	1.1%	3.9%	
**Place of residence**			
Big city ≥ 100,000	59.3%	65.3%	> 0.05
Small city < 100,000	22.5%	23%	
Town 500–3000	9.9%	7.4%	
Village < 500	8.3%	4.3%	

*Note* BMI– body mass index; p– the level of marginal significance within a statistical hypothesis test

Walking time, MVPA, and SB data for men and women are presented in Table 2. There was no significant difference between men and women in regard to walking time, and we observed that both men and women spent an equal amount of time on walking. The survey revealed that the total MVPA was higher in men than in women (*p* < 0.0001). In addition, for men the MVPA was higher both at work (*p* < 0.015) and on weekends (*p* < 0.001) than for women. The length of SB time was similar for men and women.

**Table 2:** Average (σ) descriptive data of lifestyle parameters

Parameter	Gender		p-value	$\eta_{p}^{2}$
	Female	Male		
Walking at work, min/week	356 (103.5)	398.8 (125.8)	0.378	0.001
Walking to work and back home, min/week	389.5 (89.8)	367.1 (118.5)	0.541	0.001
Leisure walking, min/week	332.6 (91.3)	251.8 (88.1)	0.038	0.001
Total walking time, min/week	1078.1 (284.6)	1017.7 (332.4)	0.39	0.001
MVPAw, min/week	325.8 (98.4)	438.4 (126.4)	0.015	0.001
MVPAh, min/week	286.9 (101.1)	259.8 (88.9)	0.49	0.001
MVPAlt, min/week	174.2 (71.2)	358.4 (88.6)	0.0001	0.07
MVPA total, min/week	786.9 (270.7)	1056.6 (303.9)	0.0001	0.03
SBw, min/d	309.5 (99.5)	313.2 (103.7)	0.73	0.001
SBlt, min/d	237.9 (77.8)	245.9 (88.7)	0.43	0.001
SB total, min/d	547.4 (177.3)	559.1 (192.4)	0.58	0.001

*Note* MVPAw– work-related moderate to vigorous physical activity; MVPAh– household moderate to vigorous physical activity; MVPAlt- leisure-time moderate to vigorous physical activity; SBw– work-related sedentary behaviour; SBlt–leisure-time sedentary behaviour; σ– the standard deviation; p– the level of marginal significance within a statistical hypothesis test; when $\eta_{p }^{2}$ falls in the interval from > 0.01 to < 0.06, then it is considered a small effect size, when from > 0.06 to < 0.14– medium effect size

**Fig. 1:**
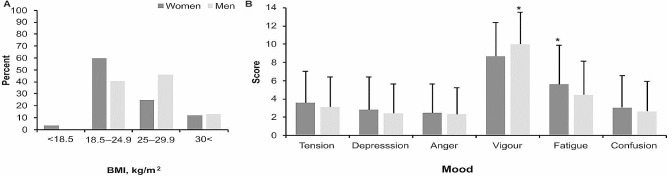
BMI structure (**A**) and the average (σ) descriptive data of mood variables (**B**). *p < 0.01 between women and men

The BMI structure in men and women is presented in Fig. 1A. There were more women than men with normal body weight (*p* < 0.0001); in addition, more men than women were overweight (*p* < 0.0001). Mood assessment revealed that men had lower subjective fatigue and higher vigour than women (*p* < 0.01; Fig. 1B).

A regression analysis revealed a significant correlation of all mood indicators with age (better results with older age), although a correlation between mood and gender was observed only for vigour and fatigue indicators (Table 3).

**Table 3:** Correlation of mood indicators with lifestyle and sociodemographic parameters

Parameter	Vigour		Fatigue		Depression		Anger		Tension		Confusion	
	β	p	β	p	β	p	β	p	β	p	β	p
MVPAw	0.042	0.207	0.036	0.282	−0.013	0.71	−0.028	0.42	0.015	0.66	0.005	0.88
MVPAh	0.032	0.298	0.039	0.201	0.004	0.88	0.001	0.99	0.001	0.97	0.019	0.55
MVPAlt	0.28	0.0001	−0.153	0.0001	−0.119	0.0001	−0.099	0.001	−0.108	0.0001	−0.093	0.001
MVPA total	0.141	0.008	−0.034	0.289	−0.024	0.81	−0.048	0.65	0.024	0.81	−0.001	0.99
Walking at work	0.054	0.072	0.036	0.245	−0.017	0.58	−0.002	0.97	0.029	0.385	−0.016	0.65
Walking to work and back	0.148	0.0001	−0.105	0.003	−0.097	0.006	−0.085	0.017	−0.078	0.03	−0.059	0.09
Leisure walking	0.043	0.065	−0.044	0.095	0.013	0.71	0.036	0.34	−0.003	0.94	0.018	0.623
SBw	−0.005	0.89	0.042	0.214	0.036	0.31	0.022	0.53	0.01	0.72	0.054	0.114
SBlt	−0.108	0.0001	0.098	0.001	0.095	0.003	0.077	0.026	0.14	0.001	0.137	0.0001
Gender	0.128	0.0001	−0.131	0.0001	−0.053	0.073	−0.025	0.45	−0.044	0.072	−0.037	0.084
Age	0.127	0.0001	−0.232	0.0001	−0.177	0.0001	−0.133	0.0001	−0.171	0.0001	−0.170	0.0001
Urban vs. rural	−0.027	0.339	0.007	0.88	0.011	0.74	0.013	0.66	0.006	0.83	0.01	0.72
Education	0.03	0.0131	0.026	0.381	0.003	0.92	0.004	0.89	0.025	0.42	−0.008	0.78
Job type	0.041	0.245	0.021	0.57	−0.007	0.86	0.019	0.59	0.01	0.79	0.027	0.44

*Note* MVPAw– work-related moderate to vigorous physical activity; MVPAh– household moderate to vigorous physical activity; MVPAlt- leisure-time moderate to vigorous physical activity; SBw– work-related sedentary behaviour; SBlt–leisure-time sedentary behaviour; β– the regression coefficient; p– the level of marginal significance within a statistical hypothesis test

A significant correlation of all mood indicators was observed only with leisure-time MVPA and leisure-time SB; no significant correlation was noted between mood indicators and household MVPA and work-related MVPA as well as work-related SB. There was a significant correlation between leisure-time SB and all mood indicators. In addition, all mood indicators, except for depression and confusion, correlated significantly with walking to work and back home.

We found that all mood indicators were significantly associated only with leisure-time MVPA and with leisure-time SB, whereas there was no significant relationship with household MVPA and work-related MVPA, or with work-related SB. However, leisure-time SB was significantly related to all mood indicators.

In addition, only vigour was correlated significantly with total MVPA. It is interesting that vigour was correlated with education; however, there was no significant correlation between mood indicators and urban or rural place of residence or with type of job.

According to regression analysis data, there was a significant correlation between work-related MVPA and education as well as type of job; between household MVPA and age, place of residence, and type of job; and between leisure-time MVPA and gender, type of job, and exercising (Table 4).

**Table 4:** Correlation between sociodemographic and lifestyle parameters

Parameter		Gender	Age	City vs. country	Education	Work type	Do you exercise?
MVPAw	β	0.01	−0.0011	0.011	−0.099	0.456	−0.016
	p	0.68	0.97	0.68	0.009	0.0001	0.57
MVPAh	β	−0.016	0.134	0.149	−0.008	0.087	−0.011
	p	0.61	0.0001	0.0001	0.79	0.003	0.71
MVPAlt	β	0.125	−0.051	0.002	−0.048	0.075	0.179
	p	0.002	0.117	0.95	0.11	0.01	0.0001
Walking at work	β	−0.001	0.068	0.051	−0.059	0.415	−0.01
	p	0.98	0.023	0.069	0.05	0.0001	0.75
Walking to work and back home	β	−0.03	0.048	−0.011	−0.015	0.037	0.094
	p	0.32	0.13	0.77	0.63	0.24	0.001
Leisure walking	β	−0.056	0.04	−0.033	−0.045	−0.012	0.086
	p	0.063	0.24	0.27	0.15	0.68	0.006
SBw	β	0.047	−0.131	−0.004	−0.036	−0.445	−0.079
	p	0.064	0.0001	0.89	0.192	0.0001	0.007
SBlt	β	0.038	−0.125	−0.056	0.024	−0.189	−0.145
	p	0.193	0.0001	0.055	0.47	0.0001	0.0001

*Note* MVPAw– work-related moderate to vigorous physical activity; MVPAh– household moderate to vigorous physical activity; MVPAlt- leisure-time moderate to vigorous physical activity; SBw– work-related sedentary behaviour; SBlt–leisure-time sedentary behaviour; β– the regression coefficient; p– the level of marginal significance within a statistical hypothesis test

**Fig. 2:**
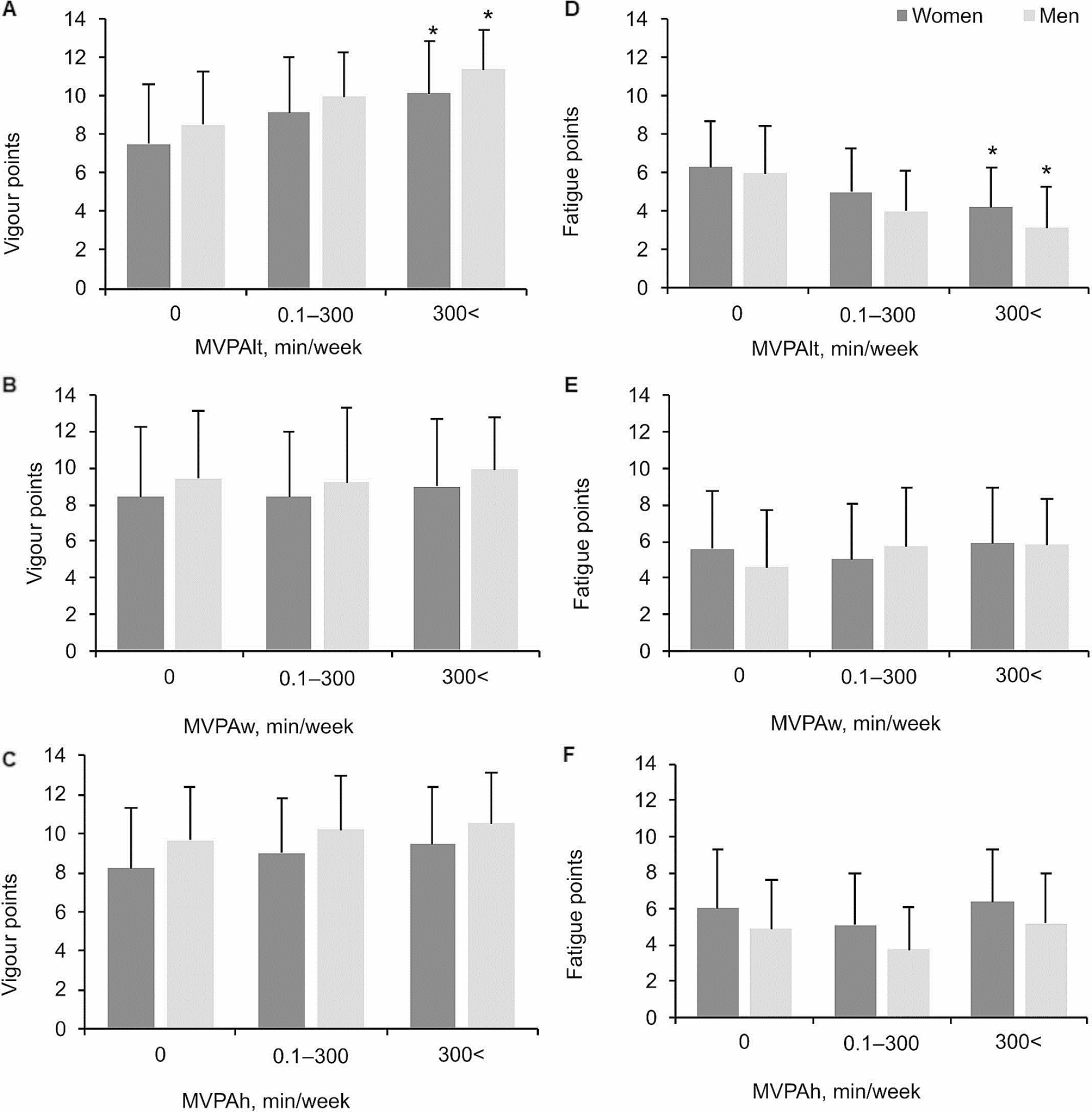
Correlation of mood variables with MVPAlt (**A**, **D**), MVPAw (**B**, **E**) and MVPAh (**C**, **F**). *p < 0.05, compared to 0 and 0–300 min/week

It should be noted that work-related SB and leisure-time SB correlated significantly with the same indicators: age, type of job, and exercising. However, leisure-time SB was more strongly related with exercising, whereas work-related SB correlated more strongly with type of job. A strong correlation was found between leisure-time MVPA and work-related MVPA (β = 0.124, *p* < 0.0001) as well as household MVPA (β = 0.123, *p* < 0.0001; correlation coefficients are adjusted to gender and age). There was a strong correlation between a sedentary workday and sedentary leisure time (β = 0.296, *p* < 0.0001, adjusted to gender and age). Leisure-time MVPA correlated strongly only with walking during leisure time (β = 0.255, *p* < 0.0001, adjusted for gender and age), whereas no significant correlation was noted with walking at work (β = 0.05, *p* = 0.106, adjusted for gender and age) and walking to work and back home (β = 0.014, *p* = 0.67, adjusted for gender and age). The survey results showed that vigour and fatigue correlated significantly only with leisure-time MVPA (two-way ANOVA for vigour: leisure-time MVPA effect: *p* < 0.0001, $\eta_{\text{p}}^{2}$= 0.12; gender effect: *p* < 0.0001, $\eta_{\text{p}}^{2}$= 0.09, ns; two-way ANOVA for fatigue: leisure-time MVPA effect: *p* < 0.0001, $\eta_{\text{p}}^{2}$= 0.09; gender effect: *p* < 0.0001, $\eta_{\text{p}}^{2}$= 0.091; ns; Fig. 2).

## Discussion

The key finding of this study is that all mood indicators correlated significantly only with leisure-time MVPA and leisure-time SB as well as walking to work and back home. The regression analysis revealed rather unexpected results, showing the absence of a statistically significant correlation between the mood indicators and work-related MVPA, household MVPA, work-related SB, walking to work and back home. Thus, mood (better mood) was related not with any kind of PA or any kind of SB but only with PA level and leisure-time SB and walking to work and back home.

This is the first study to our knowledge that has demonstrated the manifestation of the PA paradox in all mood indicators (vigour, fatigue, depression, tension, anger, confusion). It was observed in men and women and in individuals of different ages. These findings coincide with the findings of other researchers stating that health improves not only with work-related PA but also with leisure-time PA [14–16, 19, 20]. In addition, previous studies also have shown that one’s mood profile depends on PA, but those studies did not distinguish the structure of PA (i.e., neither the time when it occurred nor its intensity [7, 8]. It is known that PA improves emotional health, and previous research clearly shows that PA improves emotional intelligence [28, 29].

Our survey revealed that men had higher vigour and lower fatigue than women. These findings confirm previous findings [28, 29]. The finding that the mood profile improves with age was unexpected, although similar results have been obtained earlier [28, 29]. In addition, in our case the mood profile did not depend either on education, place of residence, or on type of job.

Leisure-time MVPA correlated significantly with gender (men had higher leisure-time MVPA level) and exercising (higher in individuals who exercise). The population-based study conducted by García-Soidán et al. sought to discover the physical activity habits of new generations and noticed controversial results, with boys being more physically active but also showing greater use of digital devices, which is typically associated with sedentary behaviour [30]. In addition, our research data showed that leisure-time SB correlated strongly with age (older people sit for a shorter time than young people), exercising, and type of job, whereas walking to work and back home correlated with exercising. We believe that people who exercise more often choose other forms of mobility. Our survey showed that work-related MVPA was mainly related to type of job and education. Naturally, people with lower education are more likely working in manual occupations with physically demanding tasks. Household MVPA correlated strongly with age and lifestyle (e.g., older people living in rural areas). Sitting at work depended on the type of job and age. According to the latest systematic literature review, leisure-time PA is negatively related to work-related PA [21]. However, we did not observe a negative correlation between leisure-time MVPA and work-related MVPA or household MVPA.

Holtermann et al. gave six reasons why work-related PA does not improve cardiovascular health, whereas leisure-time PA improves it. One of the main reasons of this PA paradox is that health-enhancing PA requires breaks, such as during leisure time [23]. Work-related PA lasts throughout the entire workday, with very short breaks for rest. However, this PA paradox is not observed worldwide. For instance, large-sample surveys in China have shown that work-related PA was not associated with mortality risk [22]. The Copenhagen City Heart Study revealed that leisure-time PA lowered systolic blood pressure, whereas work-related PA was associated with higher systolic blood pressure [19]. Therefore, the clinically significant practical implications of our study lie in the fact that it is necessary to find public health interventions aimed not only at increasing general physical activity, but also at certain corrections in the structure of physical activity, possibly specific for men and women of different ages, education, type of job, place of residence and lifestyle parameters.

## Limitations

There are some important limitations of the present study. The first limitation is that the data are based on a self-report measure of PA and the absence of objectively measured PA. The second limitation is that the sample is not representative of the entire population of Lithuania. The majority of participants in our survey had higher education and were urban residents; individuals without higher education and living in rural areas were not adequately represented. The third limitation is that our survey did not allow us to determine the exact causality between moods and the structure of PA.

## Conclusions

This study provides theoretical implications of the physical activity paradox, justifying the benefits of moderate to vigorous physical activity practiced in different circumstances. The present results show a significant positive correlation between women and men moods and leisure-time MVPA, the length of time walking to work and back home, and negative correlation between moods and leisure-time SB. However, there was no significant correlation between moods and work-related MVPA and household MVPA, walking at work, and sitting duration at work. According to the regression analysis, exercising men in all age groups moved the most (had a higher MVPA level) during leisure time, the highest work-related MVPA was observed in men and women with lower education, and the highest household MVPA was observed in older age men and women living in rural areas. Clinicians and leaders at all levels of health care should consider the greater importance of leisure-time physical activity for mental health when choosing the most targeted physical activity recommendations for mood profile improvement in men and women of different age groups. Public health policies must consider the individual differences to make these recommendations effective for specific populations, such as persons with lower education and elderly persons living in rural areas, in order to increase their physical activity during leisure time. It is likely that the practical implications of this study could help to adjust the physical activity structure of the specific populations and thus contribute to the improvement of their quality of life. Further research should focus on finding interventions for these specific groups that can increase their leisure-time physical activity.

## Data Availability

The datasets used and/or analyzed during the current study are available from the corresponding author on reasonable request.

## Abbreviations

ANOVA: analysis of variance
BMI: body mass index
MVPAh: household moderate to vigorous physical activity
MVPAlt: leisure-time moderate to vigorous physical activity
MVPAw: work-related moderate to vigorous physical activity
PA: physical activity
SB: sedentary behaviour
SBlt: leisure-time sedentary behaviour
SBw: work-related sedentary behaviour
p: the level of marginal significance within a statistical hypothesis test
β: the regression coefficient
σ: the standard deviation
$\eta_{\text{p}}^{2}$: the partial eta squared

## References

[1] Chekroud SR, Gueorguieva R, Zheutlin AB (2018). Association between physical exercise and mental health in 1·2 million individuals in the USA between 2011 and 2015: a cross-sectional study. Lancet Psychiatry.

[2] de Rezende LF, Rodrigues Lopes M, Rey-López JP (2014). Sedentary behavior and health outcomes: an overview of systematic reviews. PLoS ONE.

[3] García-Soidán JL, Leirós-Rodríguez R, Romo-Pérez V, Arufe-Giráldez V (2020). Evolution of the habits of physical activity and television viewing in Spanish children and pre-adolescents between 1997 and 2017. Int J Environ Res Public Health.

[4] Gross JJ, Uusberg H, Uusberg A (2019). Mental illness and well-being: an affect regulation perspective. World Psychiatry.

[5] Ritchie H, Roser M. Mental Health. Our world in date. 2018. Available online: https://ourworldindata.org/mental-health (accessed on 06 January 2022).

[6] Gataūlinas A (2013). Subjective well-being of Lithuanian society in the context of European Union countries.

[7] Terry PC, Skurvydas A, Lisinskiene A (2022). Validation of a Lithuanian-Language Version of the Brunel Mood Scale: the BRUMS-LTU. Int J Environ Res Public Health.

[8] Terry PC, Parsons-Smith RL, Skurvydas A (2022). Physical activity and healthy habits Influence Mood Profile clusters in a Lithuanian Population. Sustainability.

[9] Sties SW, Gonzáles AI, Netto AS (2014). Validation of the Brunel Mood Scale for cardiac rehabilitation program. Brazil J Sports Med.

[10] Brandt R, Herrero D, Massetti T (2016). The Brunel Mood Scale rating in mental health for physically active and apparently healthy populations. Health.

[11] LeUnes A, Burger J (2000). Profile of Mood States research in sport and exercise psychology: past, present, and future. J Appl Sport Psychol.

[12] Terry PC, Lane AM, Fogarty GJ (2003). Construct validity of the Profile of Mood States–adolescents for use with adults. Psychol Sport Exer.

[13] Terry PC, Parsons-Smith RL, King R (2021). Influence of sex, age, and education on mood profile clusters. PLoS ONE.

[14] Holtermann A, Hansen JV, Burr H (2012). The health paradox of occupational and leisure-time physical activity. Br J Sports Med.

[15] Richard A, Martin B, Wanner M (2015). Effects of leisure-time and occupational physical activity on total mortality risk in NHANES III according to sex, ethnicity, central obesity, and age. J Phys Act Health.

[16] Coenen P, Huysmans MA, Holtermann A (2018). Do highly physically active workers die early? A systematic review with meta-analysis of data from 193 696 participants. Br J Sports Med.

[17] Coenen P, Huysmans MA, Holtermann A (2020). Towards a better understanding of the ‘physical activity paradox’: the need for a research agenda. Br J Sports Med.

[18] Cillekens B, Lang M, van Mechelen W (2020). How does occupational physical activity influence health? An umbrella review of 23 health outcomes across 158 observational studies. Br J Sports Med.

[19] Johansson MS, Holtermann A, Marott JL (2022). The physical activity health paradox and risk factors for cardiovascular disease: a cross-sectional compositional data analysis in the Copenhagen City Heart Study. PLoS ONE.

[20] Cillekens B, Huysmans MA, Holtermann A (2022). Physical activity at work may not be health enhancing. A systematic review with meta-analysis on the association between occupational physical activity and cardiovascular disease mortality covering 23 studies with 655 892 participants. Scand J Work Environ Health.

[21] Janssen TI, Voelcker-Rehage C (2023). Leisure-time physical activity, occupational physical activity and the physical activity paradox in healthcare workers: a systematic overview of the literature. Int J Nurs Stud.

[22] Luo M, Gupta N, Holtermann A (2022). Revisiting the ‘physical activity paradox’ in a Chinese context: occupational physical activity and mortality in 142,302 urban working adults from the China Kadoorie Biobank study. Lancet Reg Health West Pac.

[23] Holtermann A, Krause N, van der Beek AJ (2018). The physical activity paradox: six reasons why occupational physical activity (OPA) does not confer the cardiovascular health benefits that leisure time physical activity does. Br J Sports Med.

[24] Dalene KE, Tarp J, Selmer RM (2021). Occupational physical activity and longevity in working men and women in Norway: a prospective cohort study. Lancet Public Health.

[25] Prince SA, Rasmussen CL, Biswas A (2021). The effect of leisure time physical activity and sedentary behaviour on the health of workers with different occupational physical activity demands: a systematic review. Int J Behav Nutr Phys Act.

[26] Craig CL, Marshall AL, Sjostrom M (2003). International Physical Activity Questionnaire: 12-country reliability and validity. Med Sci Sports Exerc.

[27] Bassett DR (2003). International physical activity questionnaire: 12-country reliability and validity. Med Sci Sports Exerc.

[28] Skurvydas A, Lisinskiene A, Lochbaum M (2021). Physical activity, stress, Depression, Emotional Intelligence, logical thinking, and overall health in a large Lithuanian from October 2019 to June 2020: age and gender differences adult sample. Int J Environ Res Public Health.

[29] Skurvydas A, Lisinskiene A, Majauskiene D (2022). What types of Exercise are best for Emotional Intelligence and Logical thinking?. Int J Environ Res Public Health.

[30] García-Soidán JL, Boente Antela B, Leirós Rodríguez R (2020). ¿Los menores españoles, en su tiempo libre, prefieren dispositivos electrónicos o actividad física?. Sportis Sci J.

